# Supplementary material for: Obligate sexual reproduction of a homothallic fungus closely related to the Cryptococcus pathogenic species complex
Source: eLife. 2022 Jun 17;11:e79114. doi: 10.7554/eLife.79114 (PMC9296135; doi:10.7554/eLife.79114)
Supplement: Figure 1—figure supplement 1—source data 1. [file elife-79114-fig1-figsupp1-data1.zip › Figure_1-figure_supplement_1-source_data_1.pdf]

CHEF gel for smaller chromosomes

(switching times of 120-260 seconds)

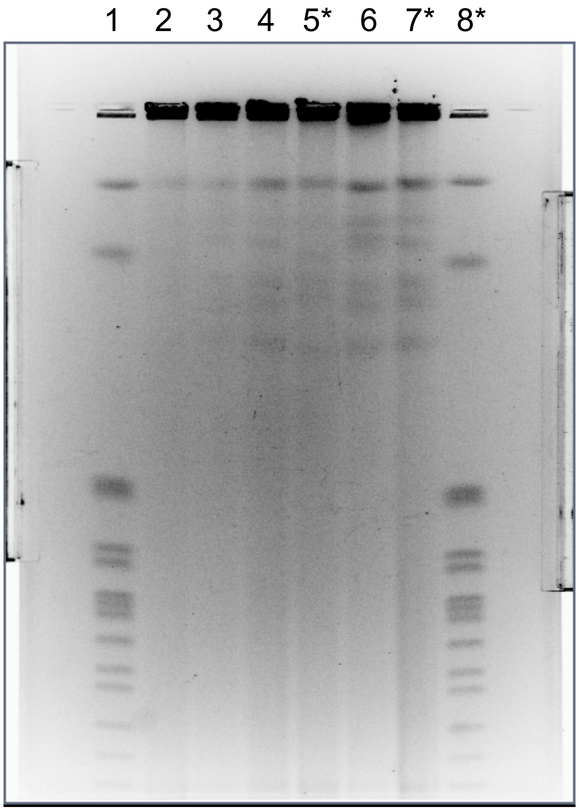

(File: FD_CHEF_SmallerChromosomes.jpg)

1 - *S. cerevisiae* ladder (Bio-Rad #1703605)
2 - *C. depauperatus* CBS7841 (1)
3 - *C. depauperatus* CBS7855 (1)
4 - *C. depauperatus* CBS7855 (2)
5* - *C. depauperatus* CBS7841 (2)
6 - *C. depauperatus* CBS7855 (3)
7* - *C. depauperatus* CBS7855 (4)
8* - *S. cerevisiae* ladder (Bio-Rad #1703605)

CHEF gel for larger chromosomes

(switching times of 560-700 seconds)

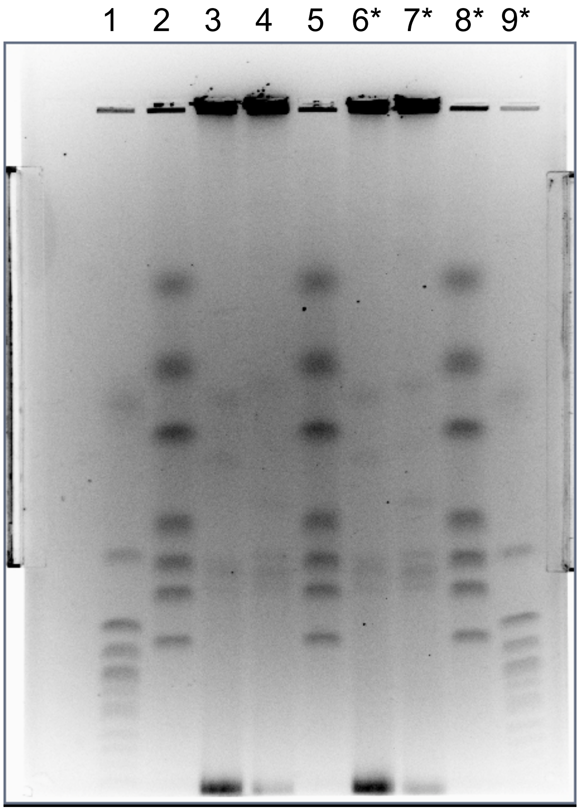

(File: FD_CHEF_LargerChromosomes.jpg)

1 - *S. cerevisiae* ladder (Bio-Rad #1703605)
2 - *H. wingei* ladder (Bio-Rad #1703667)
3 - *C. depauperatus* CBS7841 (1)
4 - *C. depauperatus* CBS7855 (1)
5 - *H. wingei* ladder (Bio-Rad #1703667)
6* - *C. depauperatus* CBS7841 (2)
7* - *C. depauperatus* CBS7855 (2)
8* - *H. wingei* ladder (Bio-Rad #1703667)
9* - *S. cerevisiae* ladder (Bio-Rad #1703605)

Lanes depicted on **Figure 1–figure supplement 1C** are indicated by asterisks in both original gels.
