# Supplementary figures and images for: Obligate sexual reproduction of a homothallic fungus closely related to the Cryptococcus pathogenic species complex
Source: eLife. 2022 Jun 17;11:e79114. doi: 10.7554/eLife.79114 (PMC9296135; doi:10.7554/eLife.79114)

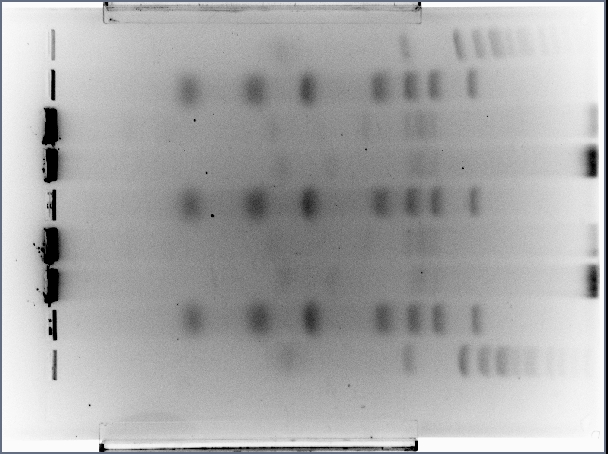

Supplement: Figure 1—figure supplement 1—source data 1. [file elife-79114-fig1-figsupp1-data1.zip › FD_CHEF_LargerChromosomes.jpg]

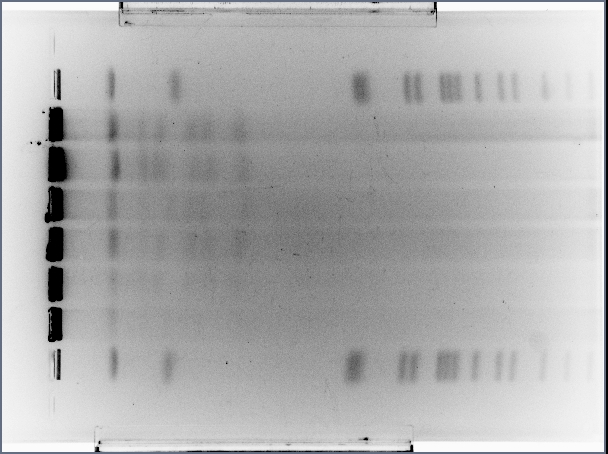

Supplement: Figure 1—figure supplement 1—source data 1. [file elife-79114-fig1-figsupp1-data1.zip › FD_CHEF_SmallerChromosomes.jpg]

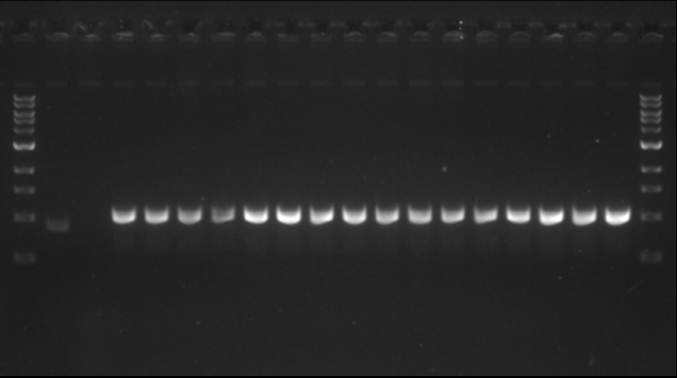

Supplement: Figure 4—source data 1. [file elife-79114-fig4-data1.zip › CnSTE6del_HPHmarkerPCR.jpg]

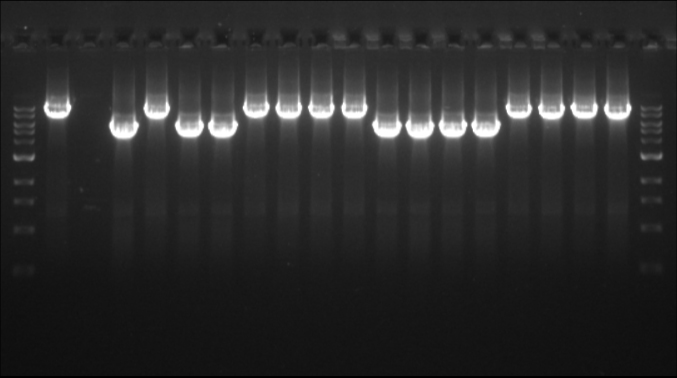

Supplement: Figure 4—source data 1. [file elife-79114-fig4-data1.zip › CnSTE6del_spanningPCR.jpg]

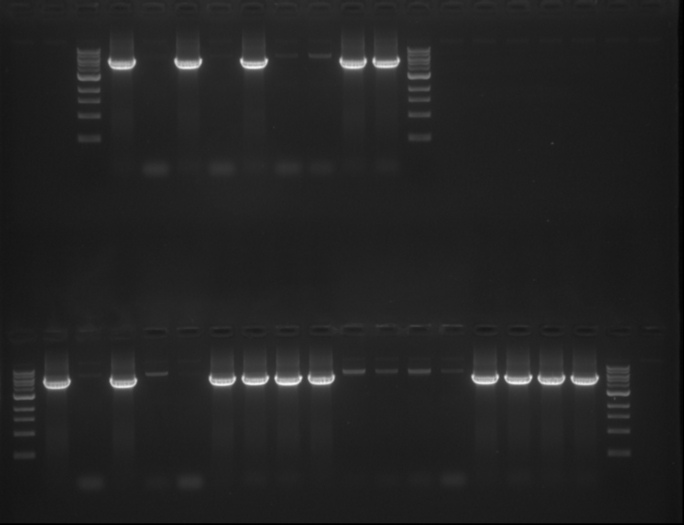

Supplement: Figure 4—source data 1. [file elife-79114-fig4-data1.zip › CnSTE6del_STE6genePCR.jpg]

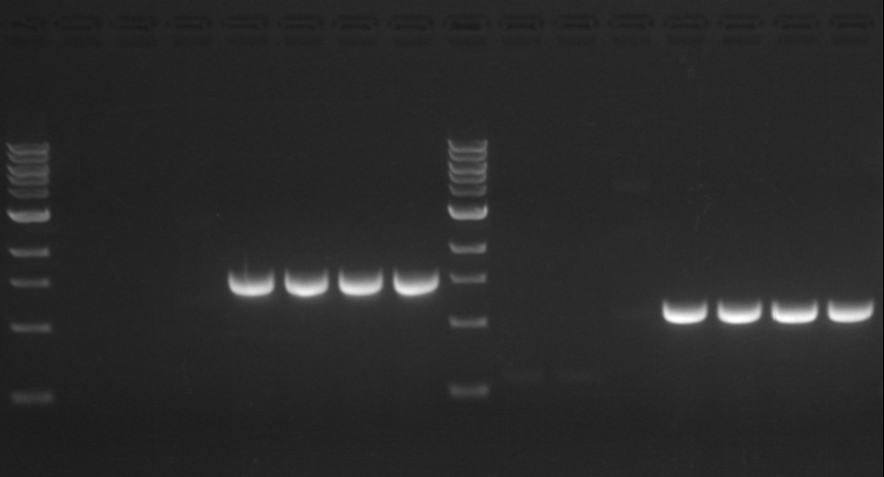

Supplement: Figure 4—source data 1. [file elife-79114-fig4-data1.zip › MFalpha_Cdep_PCRs_01.jpg]

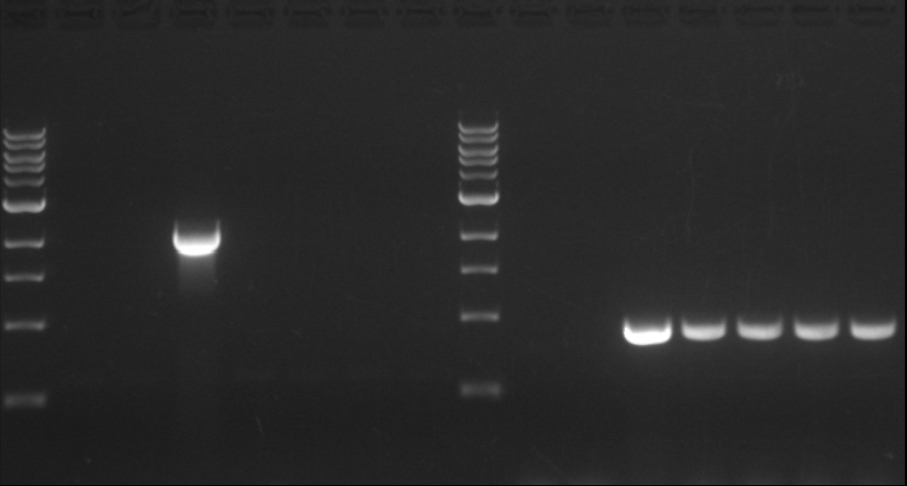

Supplement: Figure 4—source data 1. [file elife-79114-fig4-data1.zip › MFalpha_Cdep_PCRs_02.jpg]

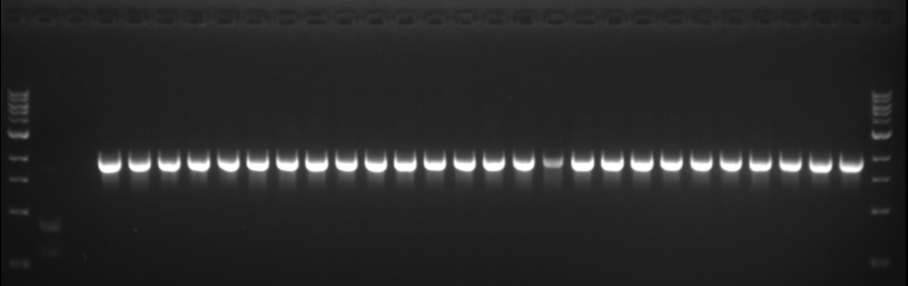

Supplement: Figure 4—source data 1. [file elife-79114-fig4-data1.zip › CnSTE3del_3junction.jpg]

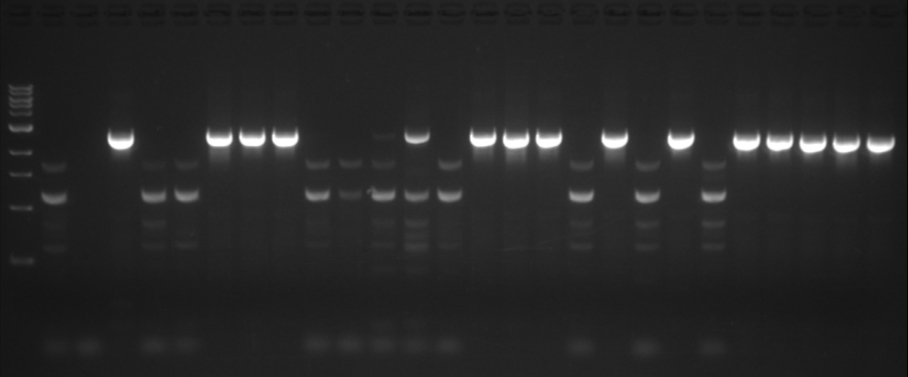

Supplement: Figure 4—source data 1. [file elife-79114-fig4-data1.zip › CnSTE3del_5junction.jpg]

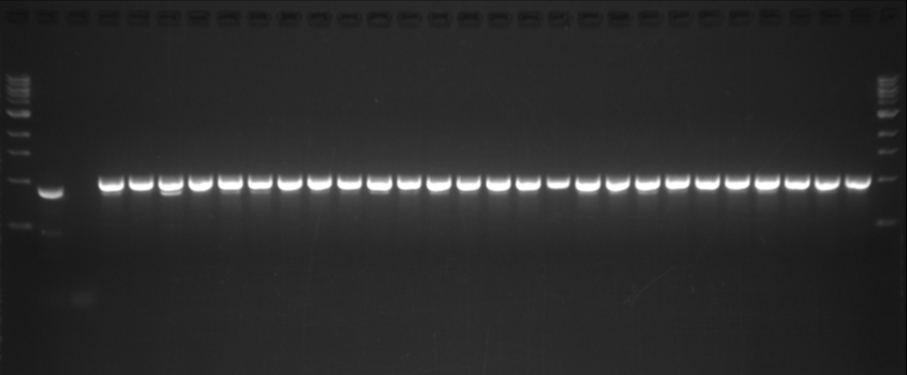

Supplement: Figure 4—source data 1. [file elife-79114-fig4-data1.zip › CnSTE3del_HPHmarkerPCR.jpg]

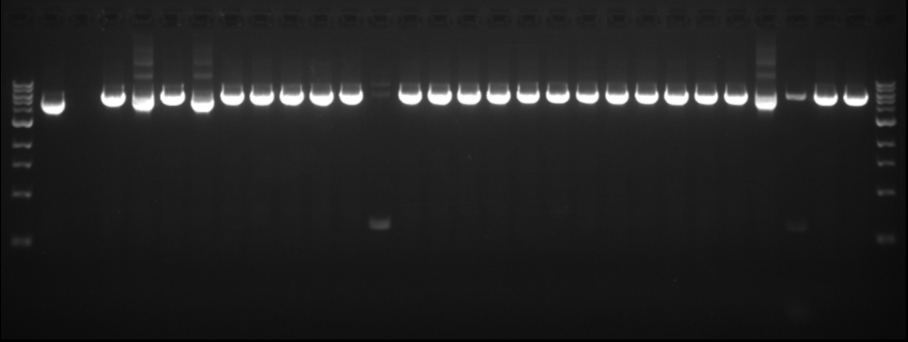

Supplement: Figure 4—source data 1. [file elife-79114-fig4-data1.zip › CnSTE3del_spanningPCR.jpg]

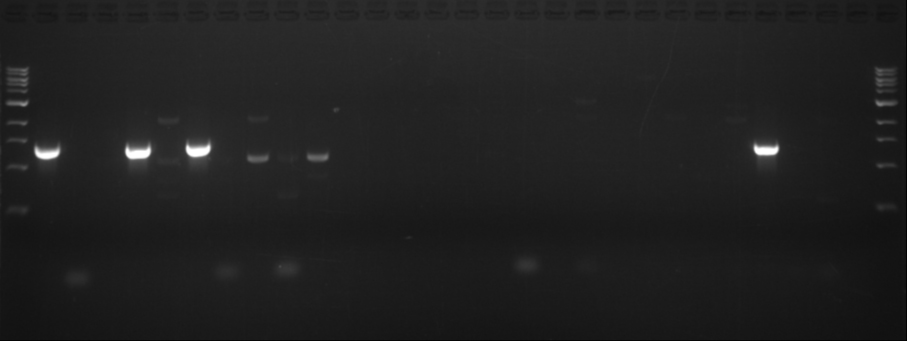

Supplement: Figure 4—source data 1. [file elife-79114-fig4-data1.zip › CnSTE3del_STE3genePCR.jpg]

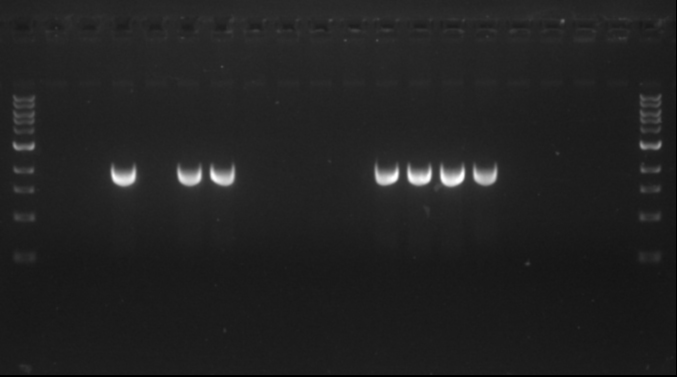

Supplement: Figure 4—source data 1. [file elife-79114-fig4-data1.zip › CnSTE6del_3junction.jpg]

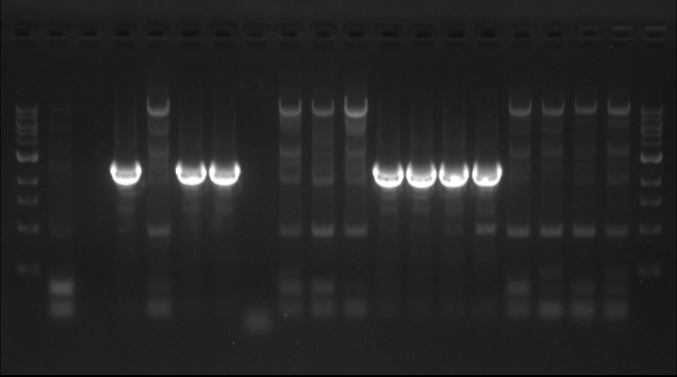

Supplement: Figure 4—source data 1. [file elife-79114-fig4-data1.zip › CnSTE6del_5junction.jpg]

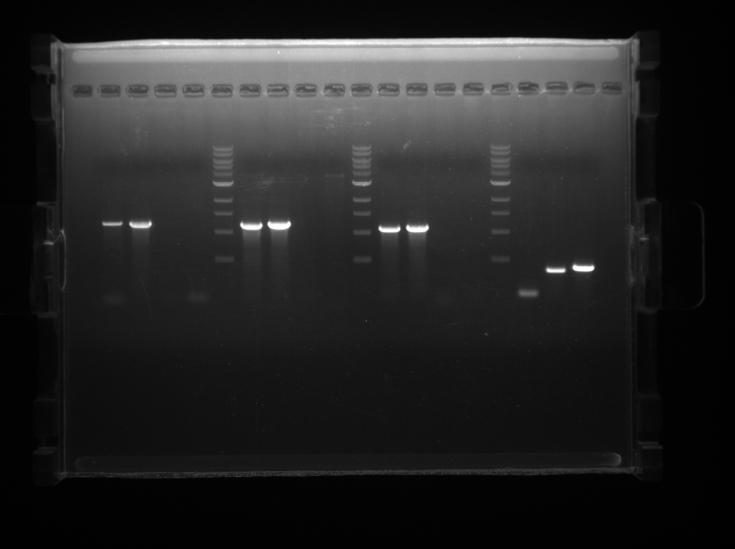

Supplement: Figure 5—figure supplement 1—source data 1. [file elife-79114-fig5-figsupp1-data1.zip › 12.23.2020_mfalpha_ste3_dmc1_mutants_all_jxns.jpg]

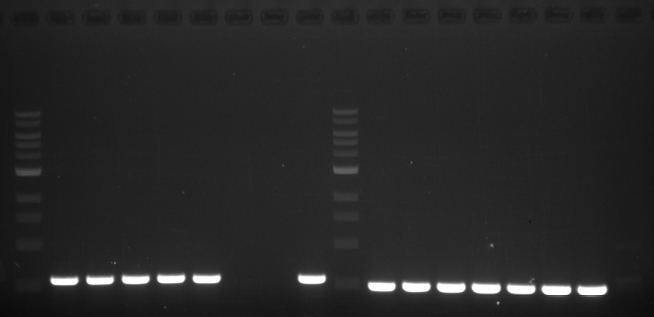

Supplement: Figure 5—figure supplement 1—source data 1. [file elife-79114-fig5-figsupp1-data1.zip › ectopic_NAT_transformants.jpg]

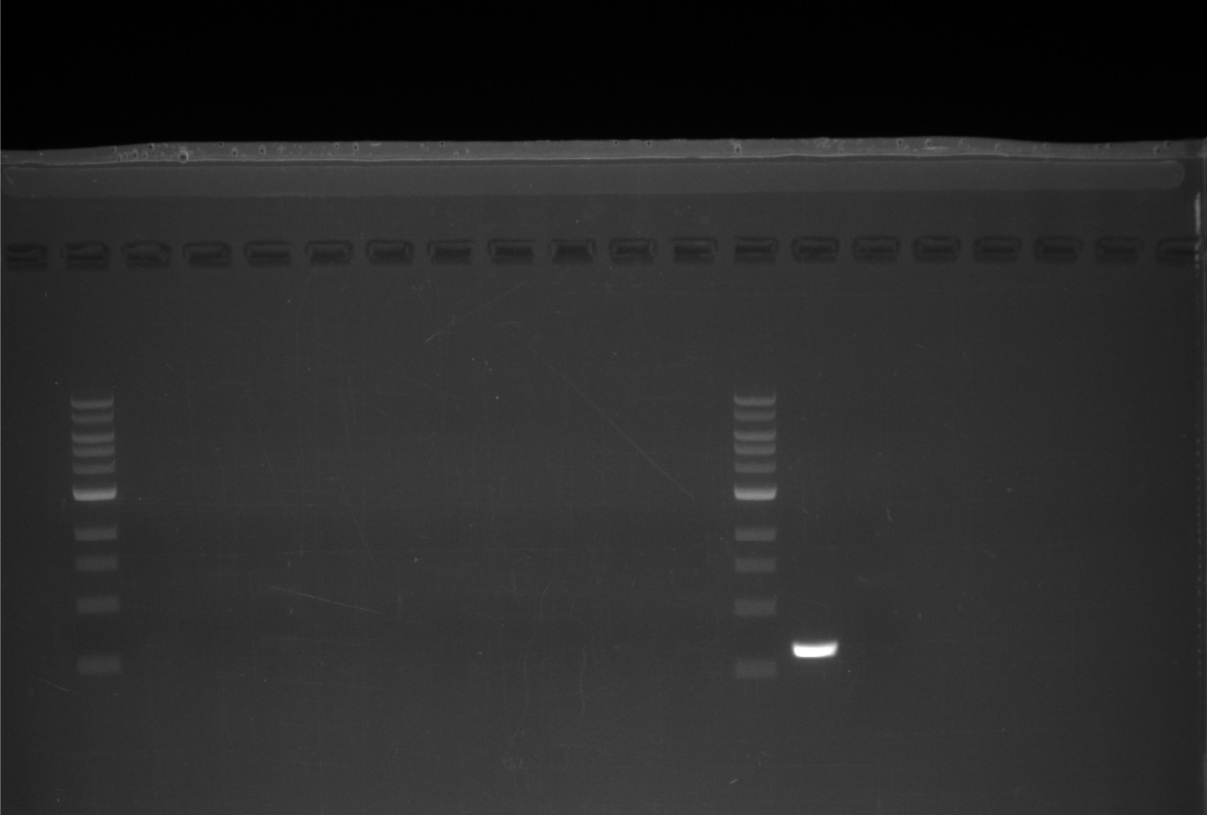

Supplement: Figure 7—source data 1. [file elife-79114-fig7-data1.zip › 2.5.19_CAN1_PCRs.jpg]

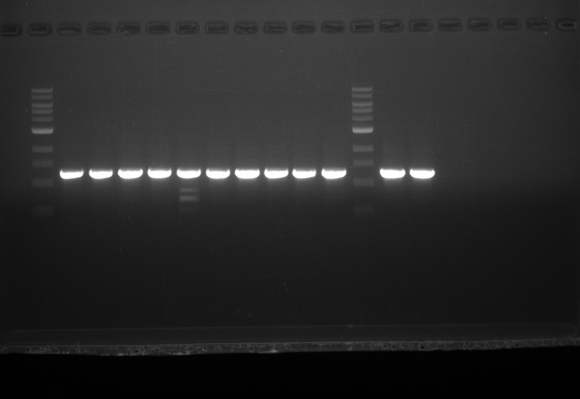

Supplement: Figure 7—source data 1. [file elife-79114-fig7-data1.zip › 2.5.19_FUR1_PCRs.jpg]

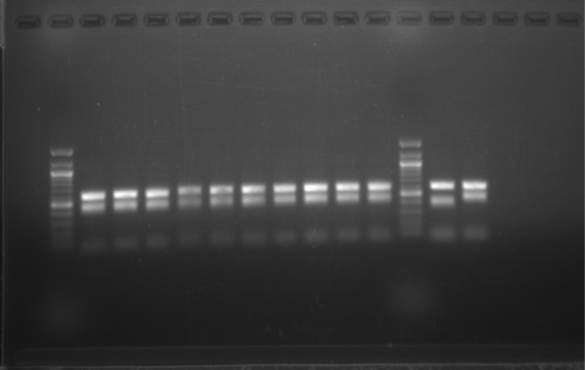

Supplement: Figure 7—source data 1. [file elife-79114-fig7-data1.zip › 2.6.19_FUR1_PCRs.jpg]

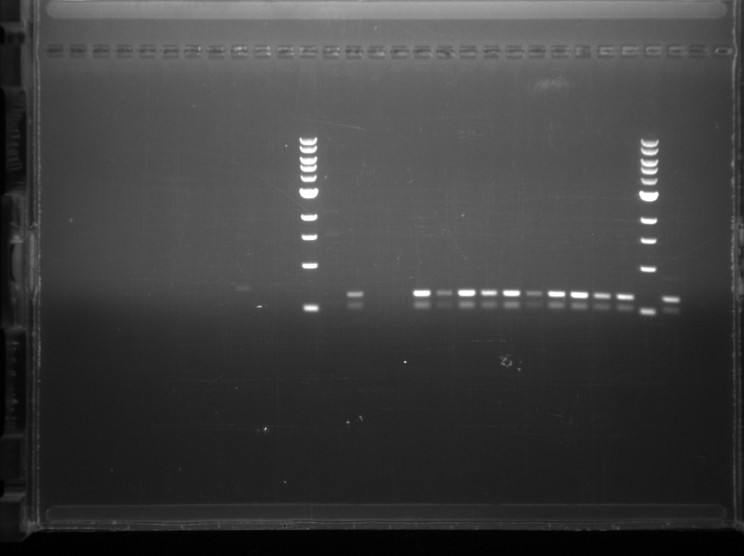

Supplement: Figure 7—source data 1. [file elife-79114-fig7-data1.zip › 3.6.2020_can1-2_PCRs.jpg]

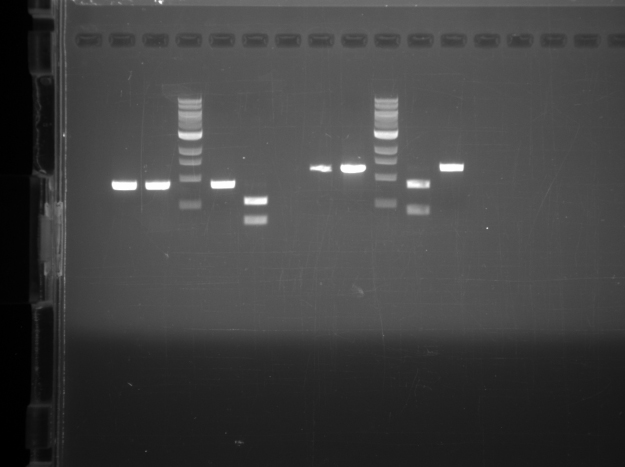

Supplement: Figure 7—source data 1. [file elife-79114-fig7-data1.zip › 3.30.18_7855_624_can1-3_and_7855_635_fur1-2_digests.jpg]
