# Supplementary material for: Obligate sexual reproduction of a homothallic fungus closely related to the Cryptococcus pathogenic species complex
Source: eLife. 2022 Jun 17;11:e79114. doi: 10.7554/eLife.79114 (PMC9296135; doi:10.7554/eLife.79114)
Supplement: Figure 4—source data 1. [file elife-79114-fig4-data1.zip › Figure4–source data_1.pdf]

► PCR validations of *C. neoformans* *MFα$_{Cd}$* transformants

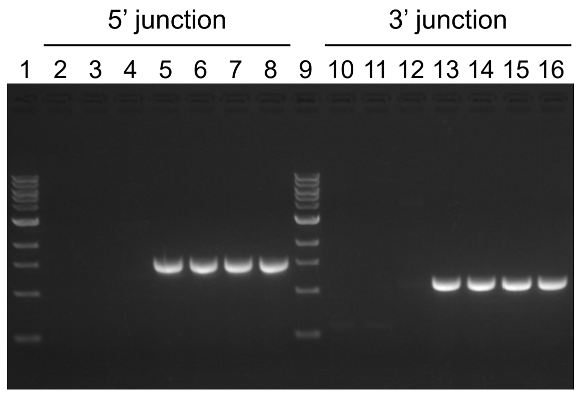

(File: MFalpha_Cdep_PCRs_01.jpg)

Note: the 5' junction and 3' junction PCRs detect the junctions between pAP1 and the safe haven insertion site.

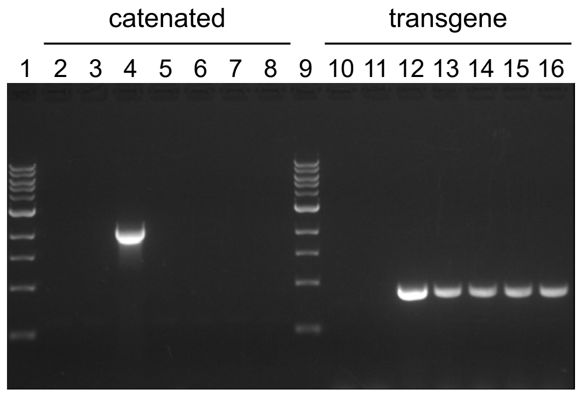

(File: MFalpha_Cdep_PCRs_02.jpg)

Note: The catenated plasmid PCR uses the 5' junction reverse primer paired with the 3' junction forward primer to detect tandem insertions. Only the plasmid (still circular) yielded a product.
The pheromone transgene PCR is specific for *MFα$_{Cd}$*.

1 - Ladder
2 - YPH570 (KN99**a** *crg1*Δ::*NAT*; parent of transformants ARP173 and ARP174)
3 - H99 crg1 (F99α *crg1*Δ::*URA5*; parent of transformants ARP242 and ARP243)
4 - pAP1 (plasmid to express *MFα$_{Cd}$* integrated at the safe haven locus)
5 - ARP173 (KN99**a** *crg1*Δ::*NAT* *MFα$_{Cd}$* NEO #1)
6 - ARP174 (KN99**a** *crg1*Δ::*NAT* *MFα$_{Cd}$* NEO #2)
7 - ARP242 (F99 *crg1*Δ::*URA5* *MFα$_{Cd}$* # 1)
8 - ARP243 (F99 *crg1*Δ::*URA5* *MFα$_{Cd}$* # 2)
9-16 - same order as before

► PCR to identify which transformants have *STE3* deleted and which ones have ectopic insertion of the *HPH (HYG)* marker gene.

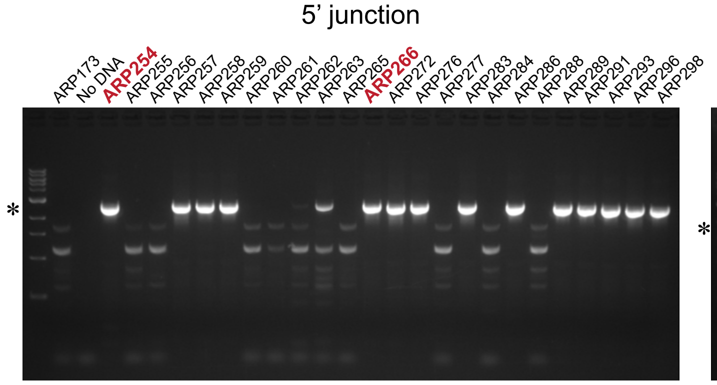

(File: CnSTE3del_5junction.jpg)

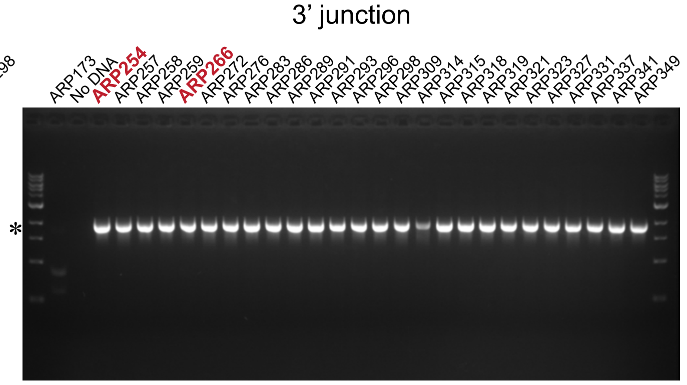

(File: CnSTE3del_3junction.jpg)

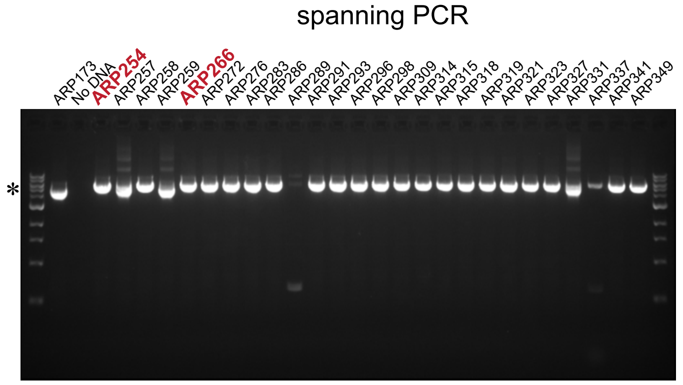

(File: CnSTE3del_spanningPCR.jpg)

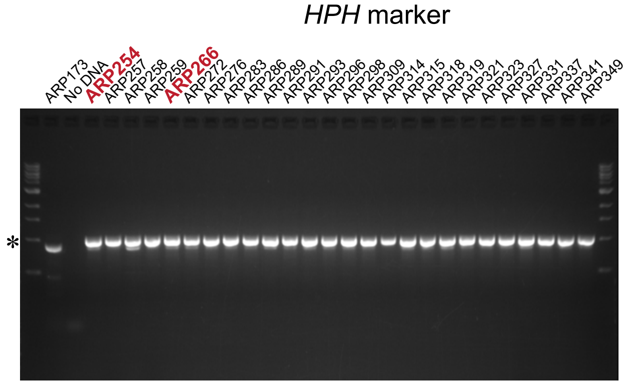

(File: CnSTE3del_HPHmarkerPCR.jpg)

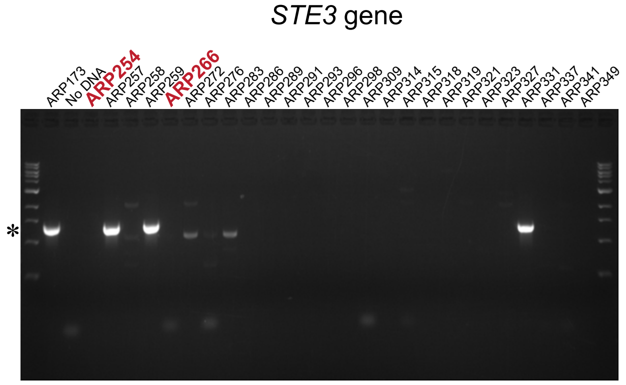

(File: CnSTE3del_STE3genePCR.jpg)

Note: correct *ste3*Δ transformants selected for further experiments (ARP254 and ARP266) are marked in red; expected band sizes in each gel are marked with an asterisk

ARP254 - KN99**a** *crg1*Δ::*NAT* *MFα$_{Cd}$* NEO *ste3*Δ::*HYG* 1.1
ARP266 - KN99**a** *crg1*Δ::*NAT* *MFα$_{Cd}$* NEO *ste3*Δ::*HYG* 2.6

► PCR to identify which transformants have *STE6* deleted and which ones have ectopic insertion of the *HPH (HYG)* marker gene.

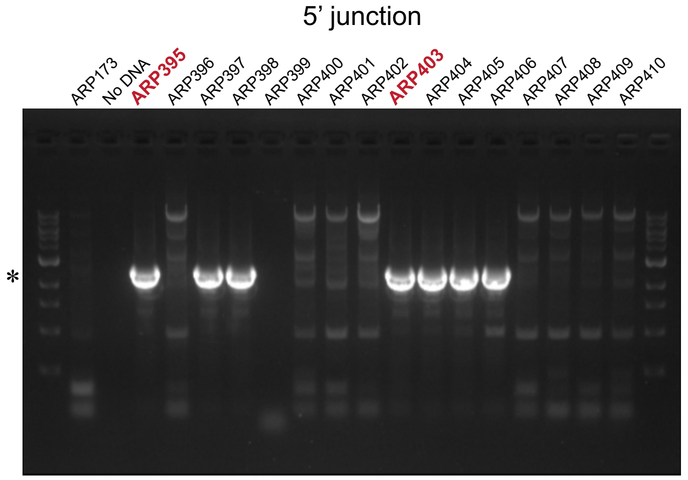

(File: CnSTE6del_5junction.jpg)

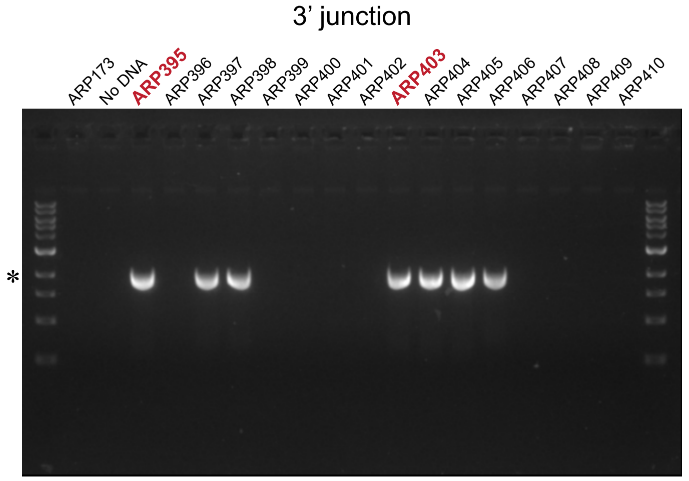

(File: CnSTE6del_3junction.jpg)

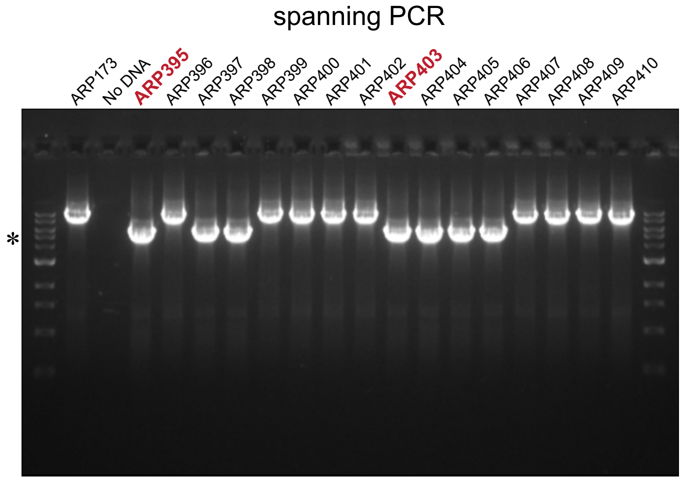

(File: CnSTE6del_spanningPCR.jpg)

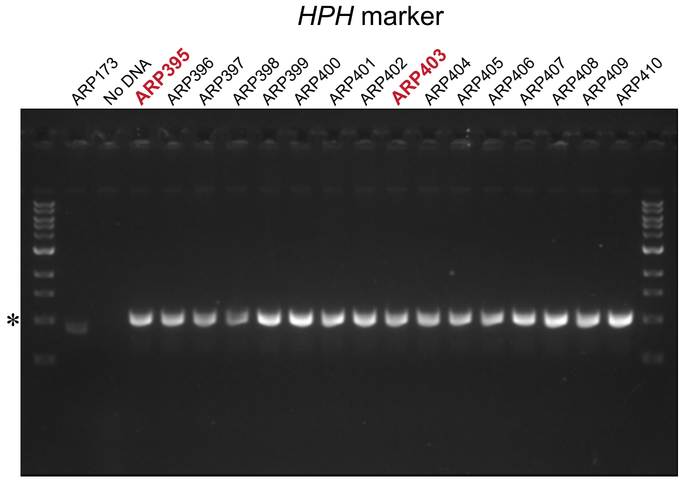

(File: CnSTE6del_HPHmarkerPCR.jpg)

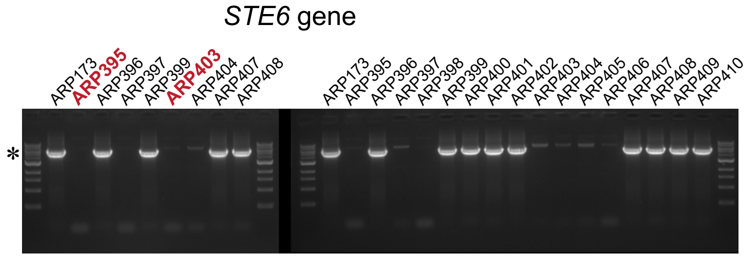

(File: CnSTE6del_STE6genePCR.jpg)

Note: correct *ste6*Δ transformants selected for further experiments (ARP395 and ARP403) are marked in red; expected band sizes in each gel are marked with an asterisk

ARP395 - KN99**a** *crg1*Δ::*NAT* *MFα$_{Cd}$* NEO *ste6*Δ::*HYG* 1.1
ARP403 - KN99**a** *crg1*Δ::*NAT* *MFα$_{Cd}$* NEO *ste6*Δ::*HYG* 2.1
