# Supplementary material for: Obligate sexual reproduction of a homothallic fungus closely related to the Cryptococcus pathogenic species complex
Source: eLife. 2022 Jun 17;11:e79114. doi: 10.7554/eLife.79114 (PMC9296135; doi:10.7554/eLife.79114)
Supplement: Figure 5—source data 1. [file elife-79114-fig5-data1.pdf]

Figure 5–source data 1. Frequency of basidia defective in sporulation (bald basidia) and basidia with spores in *C. depauperatus* wild-type (CBS7841) and mutant strains, and one-way ANOVA and Tukey's HSD post hoc statistical tests for frequencies of bald basidia.

**Strain**	**Genotype**	**Areas surveyed**	**Bald basidia**	**Basidia w/ spores**	**Total basidia**	**Frequency of bald basidia (%)**	**Frequency of basidia with spores (%)**
CBS7841	WT	1	0	25	25	0	100
		2	1	35	36	2.78	97.22
		3	1	38	39	2.56	97.44
SEC831	*mfα*Δ	1	16	3	19	84.21	15.79
		2	15	3	18	83.33	16.67
		3	11	0	11	100	0
		4	10	0	10	100	0
		5	15	0	15	100	0
		6	18	0	18	100	0
SEC836	*ste3*Δ	1	18	0	18	100	0
		2	5	0	5	100	0
		3	17	0	17	100	0
		4	16	0	16	100	0
		5	17	5	22	77.27	22.73
SEC866	*dmc1*Δ	1	39	0	39	100	0
		2	42	0	42	100	0
		3	19	0	19	100	0
#B6.1	*mfa*Δ *ste3*Δ	1	11	0	11	100	0
		2	23	0	23	100	0
		3	29	0	29	100	0
		4	5	0	5	100	0
		5	32	0	32	100	0

One-way ANOVA

Source	Degrees of freedom	Sum of Squares	Mean Square	F Ratio	p-value
Cross type	4	23656.868	5914.22	130.6550	**<.0001*****
Residuals	17	769.520	45.27		

Tukey's HSD

			95% Confidence interval		
Level	- Level	Difference	Lower	Upper	p-Value
CBS7841	*dmc1*Δ	98.21937	81.5059	114.9329	**<.0001***
CBS7841	*mfα*Δ *ste3*Δ #B6.1	98.21937	83.2704	113.1684	**<.0001***
CBS7841	*ste3*Δ	93.67392	78.7249	108.6229	**<.0001***
CBS7841	*mfα*Δ	92.81002	78.3357	107.2843	**<.0001***
*mfα*Δ	*dmc1*Δ	5.40936	-9.0650	19.8837	0.7852
*mfα*Δ	*mfα*Δ *ste3*Δ #B6.1	5.40936	-6.9857	17.8044	0.6786
*ste3*Δ	*dmc1*Δ	4.54545	-10.4036	19.4945	0.8833
*ste3*Δ	*mfα*Δ *ste3*Δ #B6.1	4.54545	-8.4008	17.4917	0.8199
*mfα*Δ	*ste3*Δ	0.86390	-11.5312	13.2590	0.9995
*mfα*Δ *ste3*Δ #B6.1	*dmc1*Δ	0.00000	-14.9490	14.9490	1.0000
