# Supplementary material for: Obligate sexual reproduction of a homothallic fungus closely related to the Cryptococcus pathogenic species complex
Source: eLife. 2022 Jun 17;11:e79114. doi: 10.7554/eLife.79114 (PMC9296135; doi:10.7554/eLife.79114)
Supplement: Figure 5—figure supplement 1—source data 1. [file elife-79114-fig5-figsupp1-data1.zip › Figure5–figure_supplement_1-source data_1.pdf]

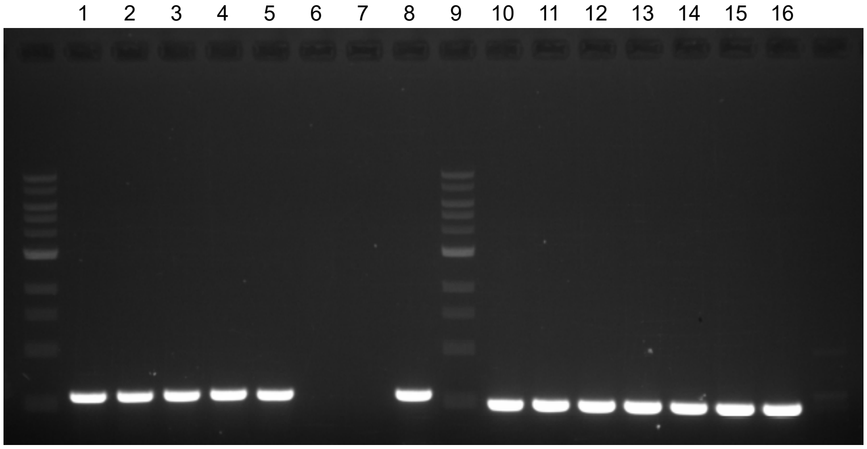

1 - CBS7841 transformant 1, *NAT* marker
2 - CBS7841 transformant 2, *NAT* marker
3 - CBS7841 transformant 3, *NAT* marker
4 - CBS7841 transformant 4, *NAT* marker
5 - CBS7855 transformant 1, *NAT* marker
6 - CBS7841 WT, *NAT* marker
7 - CBS7855 WT, *NAT* marker
8 - pSEC63, *NAT* marker
9 - Ladder
10 - CBS7841 transformant 1, *FUR1* gene
11 - CBS7841 transformant 2, *FUR1* gene
12 - CBS7841 transformant 3, *FUR1* gene
13 - CBS7841 transformant 4, *FUR1* gene
14 - CBS7855 transformant 1, *FUR1* gene
15 - CBS7841 WT, *FUR1* gene
16 - CBS7855 WT, *FUR1* gene

(File: ectopic_NAT_transformants.jpg)

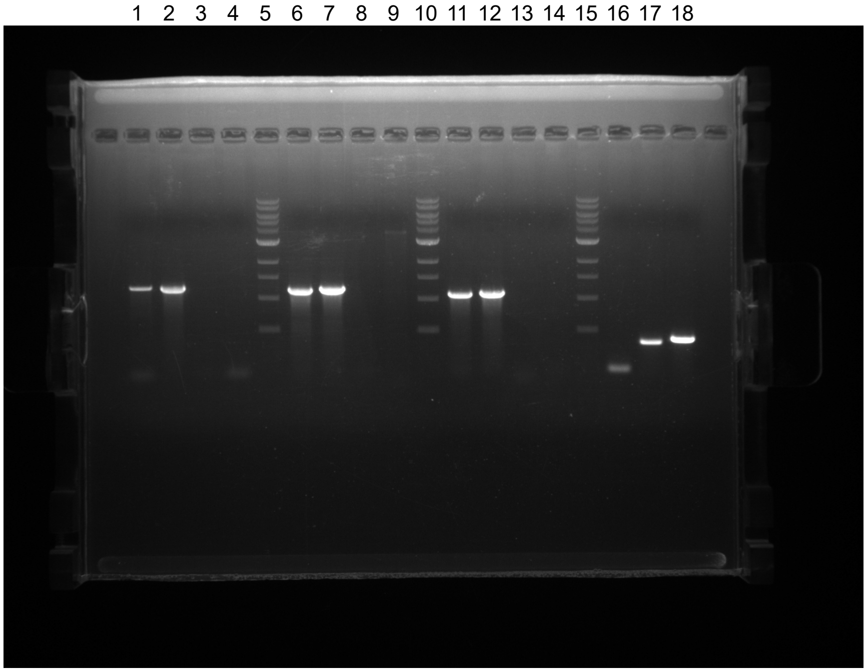

1 - *mfα*Δ::*NAT*, 5’ flanking
2 - *mfα*Δ::*NAT*, 3’ flanking
3 - *mfα*Δ::*NAT*, in-gene
4 - *mfα*Δ::*NAT*, spanning
5 - Ladder
6 - *ste3*Δ::*NAT*, 5’ flanking
7 - *ste3*Δ::*NAT*, 3’ flanking
8 - *ste3*Δ::*NAT*, in-gene
9 - *ste3*Δ::*NAT*, spanning
10 - Ladder
11 - *dmc1*Δ::*NAT*, 5’ flanking
12 - *dmc1*Δ::*NAT*, 3’ flanking
13 - *dmc1*Δ::*NAT*, in-gene
14 - *dmc1*Δ::*NAT*, spanning
15 - Ladder
16 - CBS7841 WT, *MFα*
17 - CBS7841 WT, *STE3*
18 - CBS7841 WT, *DMC1*

(File: 12.23.2020_mfalpha_ste3_dmc1_mutants_all_jxns.jpg)

Note: only the lanes depicted in **Figure 5–figure supplement 1C and 1D** are indicated (and numbered) in these original gel images.
