# Supplementary material for: Obligate sexual reproduction of a homothallic fungus closely related to the Cryptococcus pathogenic species complex
Source: eLife. 2022 Jun 17;11:e79114. doi: 10.7554/eLife.79114 (PMC9296135; doi:10.7554/eLife.79114)
Supplement: Figure 6—source data 1. — Related to Figure 6B. [file elife-79114-fig6-data1.pdf]

Figure 6–source data 1. Frequency of basidia defective in sporulation (bald basidia) and basidia with spores in *C. depauperatus* wild-type (CBS7841), *mfα*Δ and *ste3*Δ single mutants, and the *mfα*Δ x *ste3α*Δ co-cultures mutant strains and one-way ANOVA and Tukey's HSD post hoc statistical tests for frequencies of sporulating basidia. Related to Figure 6B.

**Strain**	**Genotype**	**Areas surveyed**	**Bald basidia**	**Basidia w/ spores**	**Total basidia**	**Frequency of bald basidia (%)**	**Frequency of basidia with spores (%)**
CBS7841	WT	1	2	23	25	8	92
		2	0	26	26	0	100
		3	4	26	30	13.33	86.67
		4	0	15	15	0	100
SEC831	*mfα*Δ	1	37	0	37	100	0
		2	33	1	34	97.06	2.94
		3	29	0	29	100	0
SEC836	*ste3*Δ	1	18	0	18	100	0
		2	18	0	18	100	0
		3	22	0	22	100	0
		4	39	0	39	100	0
SEC831 x SEC836	*mfa*Δ x *ste3*Δ	1	15	1	16	93.75	6.25
		2	19	1	20	95	5
		3	20	4	24	83.33	16.67
		4	25	3	28	89.29	10.71
		5	8	4	12	66.67	33.33

One-way ANOVA

Source	Degrees of freedom	Sum of Squares	Mean Square	F Ratio	p-value
Cross type	3	24028.413	8009.47	144.4004	**<.0001*** **
Residuals	12	665.605	55.47		

Tukey's HSD

			95% Confidence interval		
Level	- Level	Difference	Lower	Upper	p-Value
CBS7841	SEC836	94.66750	83.1933	106.1417	**<.0001*** **
CBS7841	SEC831	93.68750	81.2939	106.0811	**<.0001*** **
CBS7841	SEC831 x SEC836	80.27550	69.3901	91.1609	**<.0001*** **
SEC831 x SEC836	SEC836	14.39200	3.5066	25.2774	**0.0138***
SEC831 x SEC836	SEC831	13.41200	1.5615	25.2625	**0.0297***
SEC831	SEC836	0.98000	-11.4136	13.3736	0.8661
