# Supplementary material for: Obligate sexual reproduction of a homothallic fungus closely related to the Cryptococcus pathogenic species complex
Source: eLife. 2022 Jun 17;11:e79114. doi: 10.7554/eLife.79114 (PMC9296135; doi:10.7554/eLife.79114)
Supplement: Figure 7—source data 1. [file elife-79114-fig7-data1.zip › Figure7-source data1.pdf]

1 2 3 4 5 6 7 8 9 10

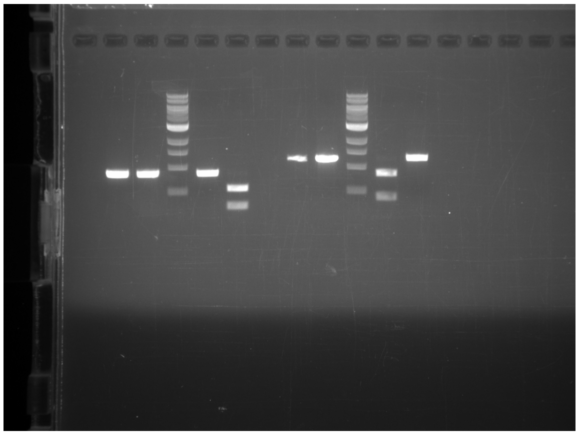

1 - CBS7855 WT
2 - CBS755 *can1-3* (SEC624)
3 - Ladder
4 - CBS7855 WT digested w/ SspI
5 - CBS755 *can1-3* (SEC624) digested w/ SspI

6 - CBS7855 WT
7 - CBS755 *fur1-2* (SEC635)
8 - Ladder
9 - CBS7855 WT digested w/ PasI
10 - CBS755 *fur1-2* (SEC635) digested w/ PasI

(File: 3.30.18_7855_624_can1-3_and_7855_635_fur1-2_digests.jpg)

1 2 3

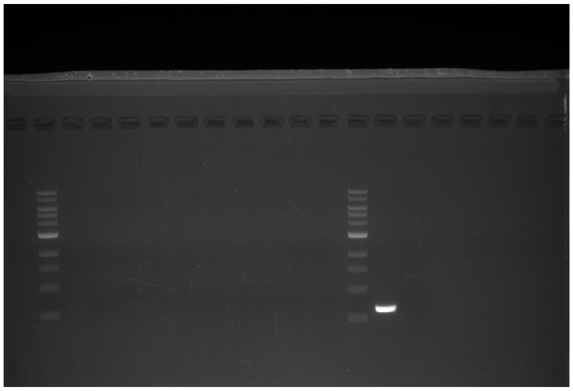

(File: 2.5.19_CAN1_PCRs.jpg)

1 - Ladder
2 - CBS7841 WT
3 - CBS7841 *can1-2* (SEC623)

123

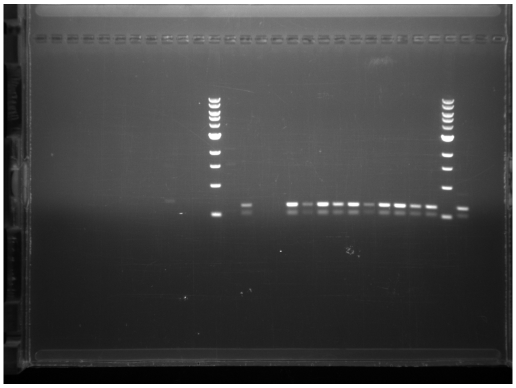

(File: 3.6.2020_can1-2_PCRs.jpg)

1 - Ladder
2 - CBS7841 WT
3 - CBS7841 *can1-2* (SEC623)

1 2 3

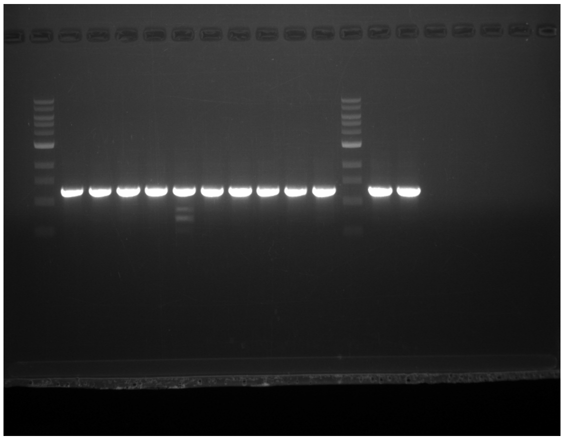

(File: 2.5.19_FUR1_PCRs.jpg)

1 - Ladder
2 - CBS7841 WT
3 - CBS7841 *fur1-1* (SEC631)

1 2 3

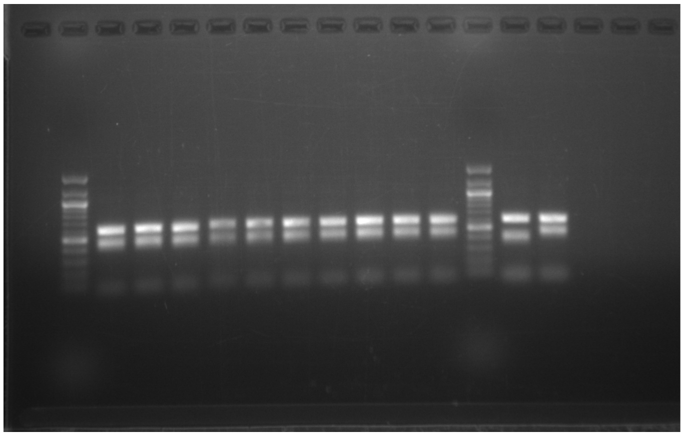

(File: 2.6.19_FUR1_PCRs.jpg)

1 - Ladder
2 - CBS7841 WT
3 - CBS7841 *fur1-1* (SEC631) digested w/ HpyCH4III

Note: only the lanes depicted in **Figure 7A and 7B** are indicated (and numbered) in these original gel images.
